# Biomechanical influence of surgical and graft‐related factors in superior capsule reconstruction: A systematic review

**DOI:** 10.1002/jeo2.70804

**Published:** 2026-06-15

**Authors:** Madalena Antunes, Carlos Quental, João Folgado, Ana Catarina Ângelo, Clara Isabel de Campos Azevedo

**Affiliations:** ^1^ IDMEC Instituto Superior Técnico, Universidade de Lisboa Lisbon Portugal; ^2^ Shoulder and Elbow Unit Hospital dos SAMS Lisbon Portugal; ^3^ Shoulder and Elbow Unit Joaquim Chaves Saúde Carcavelos Portugal

**Keywords:** biomechanical testing, cadaveric model, computational model, graft, rotator cuff tear, shoulder biomechanics, superior capsule reconstruction

## Abstract

**Purpose:**

Arthroscopic superior capsule reconstruction (ASCR) is a surgical option for the treatment of irreparable rotator cuff tears. This systematic review aimed to synthesize the available biomechanical evidence on ASCR, especially on the influence of surgical and graft‐related factors on shoulder biomechanics and graft mechanics. It was hypothesized that graft type, dimensions and concomitant procedures affect postoperative biomechanics following ASCR.

**Methods:**

A systematic review was conducted in accordance with Preferred Reporting Items for Systematic Reviews and Meta‐Analyses (PRISMA) guidelines using PubMed, Web of Science, Scopus, Cochrane Library and Ovid databases. Eligible studies included cadaveric, in vitro, in vivo and in silico biomechanical studies evaluating ASCR‐related factors. Animal studies, clinical outcome studies and comparisons with alternative techniques were excluded.

**Results:**

A total of 37 biomechanical studies were included. Investigated factors comprised concomitant procedures (29.3%), graft type (26.8%), shoulder position of fixation (22.0%), graft thickness (12.2%) and fixation technique (9.8%). Most studies used cadaveric models (86.5%) under quasi‐static conditions with simplified muscle‐loading protocols and assessed outcomes at three to five shoulder positions in the frontal plane. Most studies focused on the spacer role of the graft in limiting superior humeral head translation, while its contribution to humeral head centring and dynamic force redistribution was overlooked. Fascia lata grafts were generally associated with improved joint stability and reduced subacromial contact pressures. The shoulder position of fixation influenced joint stability, and concomitant procedures were required to restore anterior–posterior stability. Substantial heterogeneity in experimental protocols, outcome measures and reporting practices limited a direct comparison across studies.

**Conclusions:**

The current biomechanical evidence on ASCR remains constrained by methodological simplifications and the isolated, rather than combined, assessment of ASCR‐related factors. Available data suggest that graft type and dimensions, and the performance of concomitant procedures restoring capsule continuity, influence the recovery of joint stability.

**Level of Evidence:**

Basic science study: biomechanics.

**Abbreviations**
ADL: activities of the daily living
ASCR: arthroscopic superior capsule reconstruction
BAR: bursal acromial reconstruction
DOI: digital object identifier
FL: fascia lata
GH: glenohumeral
HAD: human dermal allograft
ISP: infraspinatus
LDT: latissimus dorsi tendon
LHBT: long head of the biceps tendon
LTT: lower trapezius tendon
PEEK: polyetheretherketone
PRISMA: Preferred Reporting Items for Systematic Reviews and Meta‐Analyses
PTFE: polytetrafluoroethylene
RCT: rotator cuff tear
SSC: subscapularis
SSP: supraspinatus
STT: semitendinous tendon

## INTRODUCTION

Arthroscopic superior capsule reconstruction (ASCR) was introduced by Mihata et al. [41] in 2012 for the treatment of irreparable rotator cuff tears (RCTs). In this procedure, a fascia lata (FL) graft is positioned and fixed along the physiological vector of the supraspinatus (SSP) tendon. Clinical and radiological results by Mihata et al. [37], from a 10‐year follow‐up, have shown promising long‐term outcomes regarding shoulder function and return to sports.

The ASCR is a technically demanding procedure, and several modifications have been proposed, largely motivated by the prolonged surgical time and potential donor‐site morbidity from the FL retrieval [2, 11, 20]. These modifications involve adjustments to key surgical and graft‐related factors, including changes in graft type, thickness, fixation technique, shoulder position of fixation and the performance of concomitant procedures [2, 13, 23].

The biomechanical performance of ASCR is crucial for the effectiveness of the surgical procedure, as ASCR‐related factors influence biomechanical parameters such as glenohumeral (GH) translation, subacromial contact pressure and shoulder stability. A report from 2022 documented a decline in the number of surgeons performing ASCR, citing inconsistent clinical outcomes across different combinations of surgical and graft‐related factors [25]. This decline, particularly when acellular dermal allografts were used, points out the need to define ASCR‐related factors that consistently correlate with optimal biomechanical and clinical outcomes [25, 57]. While several systematic reviews have assessed ASCR clinically, either by focusing on comparing ASCR with alternative surgical procedures [10, 26] or the use of alternative graft materials in ASCR [11, 32, 35], patient heterogeneity and confounding factors limit their ability to isolate the biomechanical contribution of each ASCR‐related factor. Biomechanical studies, using in vitro cadaveric or synthetic shoulders, robotic testing systems or computational simulations, provide controlled research of how ASCR‐related factors influence the post‐operative biomechanics of the shoulder. Despite the growing number of biomechanical studies on ASCR, the impact of surgical and graft‐related factors on shoulder biomechanics remains insufficiently synthesized.

The objective of this systematic review was to address this knowledge gap by consolidating the available biomechanical evidence, identifying which factors remain to be explored and outlining research priorities to guide the design of future biomechanical studies on ASCR. The hypothesis was that the graft type and dimensions, as well as the performance of concomitant procedures to restore capsule continuity, would influence the recovery of joint stability and kinematics.

## METHODOLOGY

This systematic review was conducted according to the PRISMA (Preferred Reporting Items for Systematic Reviews and Meta‐analyses) guidelines and was registered in the PROSPERO database [5, 42].

### Search strategy

To capture the full scope of biomechanical research on the ASCR procedure, from cadaveric to computational experiments, multiple electronic databases were selected: PubMed, Web of Science, Scopus, Cochrane Library and Ovid. The search is updated up to 26 January 2026. The search strategy was restricted to the title, abstract and keywords and combined two sets of keywords using the Boolean operator AND: (‘Superior Capsule Reconstruction’ OR ‘Superior Capsular Reconstruction’) AND (cadaver* OR biomechanic* OR robot* OR comput* OR ‘in vitro’ OR ‘in vivo’ OR ‘in silico’). Two filters were applied to include only studies published in English and from 2012 onward, consistent with the first description of the ASCR procedure by Mihata et al. [41]. The complete search queries, adapted for each electronic database, are provided in Appendix S1.

The search results, from each database, were exported to an Excel spreadsheet (Microsoft Corporation), including key bibliographic fields (authors, title, publication year, abstract and digital object identifier [DOI] when available). Duplicates were removed using automatic filtering and manually checked.

### Screening and eligibility criteria

The study selection process consisted of an initial screening of the titles and abstracts, followed by full‐text assessment of the papers that met the preliminary criteria. Eligible studies included cadaveric, in vitro, in vivo and in silico biomechanical studies evaluating ASCR‐related factors impact on shoulder biomechanics or graft mechanics. Exclusion criteria included: not addressing the shoulder joint or its biomechanics; not addressing ASCR; publication types such as editorials, errata, letters to editor, narrative reviews, systematic reviews, meta‐analyses, conference abstracts without full data, case reports or expert opinions; studies focusing only on the description of the ASCR surgical technique, patient indications and clinical outcomes; animal studies; and comparison of ASCR with other surgical procedures. Additionally, biomechanical studies that evaluated a single surgical or graft‐related combination, without comparison with other combinations of ASCR‐related factors, were excluded. For example, using a cadaveric approach to study how humeral translations are impacted by the fixation of an FL graft in ASCR, without considering, for example, comparison of results with other graft types. If full‐text papers were not accessible, the corresponding authors were directly contacted to request access. All full‐text articles were screened for eligibility by M. A., and included studies confirmed by the C. I. de. C. A. and A. C. A.

### Data extraction

The studies included in the qualitative synthesis step were processed through a customized data extraction table that summarized information (when available) regarding the authors, article title, year of publication, country where the experiments were performed, methodological approach, number of specimens, tendons affected by the irreparable RCT and their extent, concomitant procedures, graft type and thickness, fixation strategy (tensioning and shoulder position for the fixation of the graft), primary biomechanical outcomes and main conclusions. The reference list of the included papers was also screened to identify additional relevant records. The methodological quality of the cadaveric studies included in the systematic review was assessed using the Quality of Appraisal for Cadaveric Studies (QUACS) score, a validated 13‐item checklist designed for the evaluation of cadaveric research [55, 56].

## RESULTS

**Figure 1:**
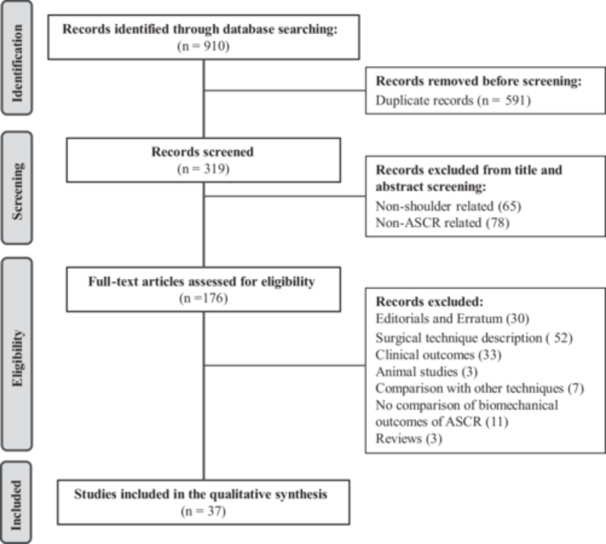
Preferred Reporting Items for Systematic Reviews and Meta‐analyses (PRISMA) flowchart. ASCR, arthroscopic superior capsule reconstruction.

The combined search across the electronic databases resulted in a total of 910 records. After removal of duplicates, 319 unique records remained for screening. Title and abstract screening resulted in the exclusion of 143 articles that were unrelated to the shoulder joint or did not address the ASCR procedure. Consequently, 176 articles were included for a full‐text assessment to determine eligibility. Among these, 30 were editorials or errata, which were examined to identify relevant references or modifications to the original text that could influence the interpretation of the biomechanical findings in the original studies. A total of 52 articles presented detailed descriptions of the ASCR surgical technique, patient indications to perform ASCR and description of surgical and graft‐related factors available in ASCR. In addition, 33 clinical studies were excluded as the focus was on patient‐reported measurements, graft integrity or post‐operative shoulder function. Three animal studies conducted in rabbits and rat models were also excluded. Seven studies were excluded because they evaluated the biomechanical performance of ASCR in comparison to alternative techniques (e.g., tendon transfers, subacromial spacer and reverse total shoulder arthroplasty). Another 11 studies were excluded because they studied the biomechanical impact of ASCR using a single configuration of surgical or graft‐related factor. These studies evaluated the role of the graft in shoulder biomechanics using different graft materials, including FL grafts, acellular dermal allografts, patellar tendon and peroneus longus tendon. Despite providing valuable insights into the biomechanics of the shoulder after ASCR, they do not allow a direct comparison of surgical or graft‐related parameters, as the experimental set‐up differed between studies, thereby limiting their inclusion in a comparative analysis. Finally, three published review articles addressing the impact of the selection of surgical and graft‐related factors on the biomechanics of the shoulder after ASCR were removed. Of these, Zhao et al. [58] and Makovicka et al. [35] integrated both clinical and biomechanical outcomes to compare graft types from clinical and biomechanical studies, whereas Ting et al. [53] focused on the impact of surgical (which did not represent the scope of options available) and patient factors on clinical outcomes. After applying all exclusion criteria, a total of 37 biomechanical studies met the inclusion criteria. The flowchart summarizing the methodology conducted in the study selection process is summarized in Figure 1. A detailed list of the excluded studies assessed for eligibility, including the respective exclusion criteria, is provided in Appendix S2.

The 37 studies were included in the qualitative synthesis and evaluated several surgical and graft‐related factors that may influence the performance of ASCR. Three types of methodological approaches were used to study the ASCR‐related factors: in vitro cadaveric (86.5%), referred hereafter as only cadaveric; in vitro synthetic (5.4%); and computational (8.1%). The surgical and graft‐related factors were grouped in five categories: concomitant procedures (29.3%), graft type (26.8%), shoulder position of fixation (22.0%), graft thickness (12.2%) and fixation technique (9.8%). Four studies focused on two surgical and/or graft‐related factors at the same time: Antunes et al. [7] evaluated shoulder position of fixation and concomitant procedures, Cline et al. [15] evaluated graft type and thickness, Mihata et al. [38] evaluated graft thickness and shoulder position of fixation, and Ting et al. [52] evaluated graft type and fixation technique. Among the cadaveric studies, 25 studies indicated excellent methodological quality (score > 81%), and the remaining 7 were classified with substantial methodological quality (score from 61% to 80%).

The following sub‐sections provide a detailed synthesis of the findings associated with each category of the evaluated ASCR‐related factors, emphasizing the differences between methodological approaches, the main biomechanical outcomes reported, consistent trends, methodological limitations and research gaps on the biomechanics of the shoulder after ASCR. These results are summarized in Table 1.

**Table 1:** Summary of the included studies in the systematic review related to ASCR‐related factors.

Author (publication year)	ASCR‐related factor studied	Methodological approach	RCT type	Graft type	Graft thickness (mm)	Shoulder position of fixation	Concomitant procedure	Muscle loading	QUACS score, %
Mihata et al. (2016) [39]	Concomitant procedure	Cadaveric	Full SSP	FL	8	30° abduction	Acromioplasty (with and without)	Static loading	92
Labib et al. (2022) [31]	Concomitant procedure	Cadaveric	SSP (Full and 50%)	NA	NA	NA	BAR (with and without)	Static loading	62
Krishnan et al. (2023) [29]	Concomitant procedure	Cadaveric	SSP (Full and 50%)	HDA	3	30° abduction	BAR (with and without)	Static loading	85
Omid et al. (2019) [44]	Concomitant procedure	Cadaveric	SSP + 50% ISP	HDA	2	20° abduction	LDT transfer (with and without)	Static loading	92
Berthold et al. (2021) [8]	Concomitant procedure	Cadaveric	SSP + 50% ISP	STT	3–4	30° abduction	LHBT tenodesis (with and without)	Dynamic loading (SSP and deltoid)	92
Lee et al. (2023) [34]	Concomitant procedure	Cadaveric	Full SSP + ISP	FL	Double‐folded	30° abduction	LTT transfer (with and without)	Static loading	92
Amirouche et al. (2025) [4]	Concomitant procedure	Cadaveric	Full SSP + ISP	HDA	NA	NA	LTT transfer (with and without)	Dynamic loading	85
Mungalpara et al. (2025) [43]	Concomitant procedure	Cadaveric	Full SSP + ISP	HDA	5	30° abduction	LTT transfer (with and without)	Dynamic loading	92
Mihata et al. (2016) [40]	Concomitant procedure	Cadaveric	Full SSP	FL	8	30° abduction	Side‐to‐side suturing (with and without)	Static loading	92
Han et al. (2019) [24]	Concomitant procedure	Cadaveric	Full SSP	LHBT	5–8.2^a^	30° abduction	Side‐to‐side suturing (with and without)	Static loading	77
Curtis et al. (2020) [16]	Concomitant procedure	Cadaveric	Full SSP	HDA	3	30° abduction	Subacromial resurfacing (with and without)	Static loading	85
Mihata et al. (2017) [36]	Graft type	Cadaveric	Full SSP	FL and HDA	FL: 8	20° abduction	Side‐to‐side suturing	Static loading	92
					HDA: 3				
Shah et al. (2021) [49]	Graft type	Cadaveric	Full SSP	FL and HDA	FL: 8	30° abduction	Side‐to‐side suturing	Static loading	92
					HDA: 6				
El‐shaar et al. (2018) [22]	Graft type	Cadaveric	Full SSP + ISP	FL and LHBT	NA	30° abduction	Side‐to‐side suturing of the FL	No muscle loading	69
Krishnan et al. (2023) [30]	Graft type	Cadaveric	Full SSP	HDA and LHBT	3	30° abduction	LHBT tenodesis	Static loading	85
de C. Azevedo et al. (2021) [12]	Graft type	Cadaveric	No muscles	Proximal and mid‐thigh FL	4.17–9.14	No graft fixation	No concomitant procedures	Load‐to‐failure	92
Lee et al. (2020) [33]	Graft type	*in vitro* synthetic (Sawbones)	No muscles	HDA (suture‐tape reinforcement)	NA	No graft fixation	No concomitant procedures	Load‐to‐failure	NA
Berthold et al. (2021) [9]	Graft type	Cadaveric	SSP + 50% ISP	V‐shaped, box‐shaped and single‐stranded LHBT	4–6	30° abduction	No concomitant procedures	Dynamic loading (SSP and deltoid)	92
Denard et al. (2021) [18]	Graft type	Cadaveric	SSP + 50% ISP	Box‐shaped and single‐stranded LHBT	5–8.2^a^	20° abduction 30° external rotation	Side‐to‐side suturing	Static loading	85
Wegmann et al. (2024) [54]	Graft type	Cadaveric	Full SSP	Narrow and wide box‐shaped LHBT	5–8.2^a^	30° abduction	NA	Static loading	77
Antunes et al. (2024) [6]	Shoulder position of fixation	Computational	Full SSP and Full SSP + (ISP and/or SSC)	FL	5	45 combinations (abduction, flexion, axial rotation)	No concomitant procedures	Dynamic loading	NA
Adams et al. (2020) [1]	Shoulder position of fixation	Cadaveric	Full SSP	HDA	2.5–3.5	0°, 15°, 30°, 45° and 60° abduction	Side‐to‐side suturing	Dynamic loading (SSP and deltoid)	85
Dyrna et al. (2020) [21]	Shoulder position of fixation	Cadaveric	Full SSP	HDA	3.5	30° abduction (with and without tensioning)	Side‐to‐side suturing	Dynamic loading (SSP and deltoid)	85
Tibone et al. (2022) [51]	Shoulder position of fixation	Cadaveric	SSP + 50% ISP	HDA	3	20° and 40° abduction	Side‐to‐side suturing	Static loading	77
Altintas et al. (2023) [3]	Shoulder position of fixation	Cadaveric	Full SSP + ISP	HDA	3	30° and 45° abduction	Side‐to‐side suturing	Static loading	85
Hast et al. (2018) [27]	Shoulder position of fixation	Computational	No muscles	HDA	1.8–3.9	64000 combinations (abduction, flexion and axial rotation)	No concomitant procedures	No muscles	NA
Paccot et al. (2025) [45]	Shoulder position of fixation	Cadaveric	SSP + 50% ISP	LHBT (anterior, middle and posterior fixation)	5–8.2^a^	0° and 30° abduction	NA	Static loading	69
Hu et al. (2025) [8]	Graft thickness	Cadaveric	SSP + 50% ISP	FL	2‐ and 4‐fold	30° abduction	Side‐to‐side suturing	Dynamic loading (deltoid)	92
Scheiderer et al. (2020) [47]	Graft thickness	Cadaveric	Full SSP	HDA	3 and 6	30° abduction	Posterior suturing	Dynamic loading (SSP and deltoid)	92
Smith et al. (2022) [50]	Graft thickness	Cadaveric	Full SSP	HDA	Single and double layer	NA	Posterior suturing	Static loading	77
Schon et al. (2017) [48]	Fixation technique	Cadaveric	No muscles	No graft	No graft	No graft	No concomitant procedures	No muscles	85
Pogorzelski et al. (2018) [46]	Fixation technique	Cadaveric	No muscles	HDA	3	No humeral fixation	No concomitant procedures	Load‐to‐failure	92
Dasari et al. (2024) [17]	Fixation technique	Cadaveric	No muscles	No graft	No graft	No graft	No concomitant procedures	Load‐to‐failure	85
Antunes et al. (2021) [7]	Shoulder position of fixation + Concomitant procedure	Computational	Full SSP	FL	5	45 combinations (abduction, flexion, axial rotation)	LHBT tenotomy	Dynamic loading	NA
Cline et al. (2021) [15]	Graft type + thickness	Cadaveric	SSP + 50% ISP	FL and HDA	Single and double layer	20° abduction	Side‐to‐side suturing	Static loading	92
Mihata et al. (2016) [38]	Graft thickness + Shoulder position of fixation	Cadaveric	Full SSP	FL	4 and 8	10° and 30° abduction	Side‐to‐side suturing	Static loading	92
Ting et al. (2023) [52]	Graft type + fixation technique	In vitro synthetic (Sawbones)	No muscles	HDA and PTFE	HDA: 1.27–1.78 PTFE: 2.87	No humeral fixation	No concomitant procedures	Load‐to‐failure	NA

Abbreviations: ASCR, arthroscopic superior capsule reconstruction; BAR, bursal acromial reconstruction; FL, fascia lata; HDA, human acellular dermal allograft; ISP, infraspinatus; LDT, latissimus dorsi tendon; LHBT, long head of the biceps tendon; LTT, lower trapezius tendon; NA, not available; PTFE, polytetrafluoroethylene; QUACS, quality appraisal for cadaveric studies; RCT, rotator cuff tear; SSC, subscapularis; SSP, supraspinatus; STT, semitendinosus tendon.

a Average thickness of the LHBT [19].

### Concomitant procedures

Three studies addressed the consequences of acromion impingement on the graft and how it can promote its early failure [29, 31, 39]. Mihata et al. [39] reported that performing acromioplasty, in the setting of a full SSP tear with an FL graft, reduced the subacromial contact area by 21% at 30° of abduction and by 61% at 60° of abduction, without increasing contact pressure. Another approach is to modify the acromial undersurface or bursal space by performing a bursal acromial reconstruction (BAR) or subacromial resurfacing. The BAR procedure was reported by Labib et al. and Krishnan et al. [29, 31] to increase the moment arms of the teres minor and subscapularis (SSC), which could promote the ability to perform abduction at higher degrees. For the fixation of an additional acellular human dermal allograft (HDA) layer, between the humeral head and acromion, Curtis et al. [16] observed reduced humeral head translations, relative to the intact condition, at the cost of increased subacromial contact pressure.

Tendon transfers were tested in the presence of superior or posterosuperior irreparable tears. Omid et al. [44] reported that the ASCR, with HDA graft, combined with latissimus dorsi tendon (LDT) transfer, improved GH stability within low to mid‐ range degrees of abduction (0° and 30°) and reduced internal rotations of the humerus measured at 60° of abduction. Concomitant transfers of the lower trapezius tendon (LTT) in ASCR, with a FL or HDA graft, consistently reduced humeral head translations and subacromial peak pressures, from 0° to 60° abduction, in the cadaveric studies by Amirouche et al., Lee et al. and Mungalpara et al. [4, 34, 43]. The role of the LHBT tenodesis or tenotomy in shoulder stability was studied experimentally and computationally by Berthold et al. and Antunes et al. [7, 8], respectively. Despite different tear patterns, graft materials and the models used to test, both studies suggest that the presence of the LHBT is required to maintain joint kinematics and stability.

Mihata et al. [40] showed that for a full SSP tear and ASCR using FL graft, the side‐to‐side suturing to the infraspinatus (ISP) and SSC completely restored intact capsule levels at 0°, 30° and 60° of GH abduction. In the cadaveric study by Han et al. [24], performing concomitant side‐to‐side suturing on a ASCR using LHBT did not produce significant improvement regarding translations of the humeral head, but the contact area with the acromion reduced to 51.6% of the intact condition at 60° of GH abduction.

### Graft type

Comparisons between proximal and mid‐thigh FL grafts, performed by de C. Azevedo et al. [12], revealed no significant differences in material properties, supporting flexibility in donor‐site selection. Other grafts have been studied to avoid the potential donor‐site morbidity and standardize the final construct mechanical properties. These include HDA, LHBT and synthetic constructs, such as polytetrafluoroethylene (PTFE). The biomechanical studies used the FL or HDA grafts as the reference condition when testing other graft types.

Compared with FL autografts, Mihata et al. [36] reported that a single‐layer HDA graft partially restored GH stability, whereas the FL grafts completely restored the native condition. Cline et al. and Shah et al. [15, 49] showed that increasing HDA thickness (double‐layer configuration) yielded a performance closer to the FL condition and was able to restore stability and decrease subacromial pressure. These results suggest that the thicker HDA may propose an alternative to FL graft; however, Mihata et al. [36] showed that HDA had greater elongation tendencies under cyclic loading, of about 15% of the initial length. Reinforcement strategies, such as suture‐tape reinforcement, have been proposed for this limitation and Lee et al. [33] showed decreased allograft elongation and maintenance of graft thickness.

The LHBT has emerged as a graft material alternative. Cadaveric studies by El‐shaar et al. and Krishnan et al. [22, 30] showed that ASCR using the LHBT as a graft to restore superior stability showed biomechanically equivalent results compared to the FL and HDA grafts. However, the configuration of the LHBT graft has been shown, by Berthold et al. and Denard et al. [9, 18], to impact superior translations and subacromial contact pressures and areas. Box‐shaped and V‐shaped configurations significantly reduced superior translations compared to a single‐stranded LHBT graft [9, 18]. A narrow box‐shaped LHBT graft was shown, by Wegmann et al. [54], to be biomechanically superior to a wide box‐shaped configuration.

Synthetic materials have also been tested in in vitro synthetic approaches using Sawbones (anatomical models of the human bones). Ting et al. [52] reported that PTFE reached higher yield loads compared to HDA under uniplanar cyclic loading; for example, using two anchors and suture, the PTFE reached mean yield loads of 268N, while the HDA only reached mean values of 194N.

### Shoulder position of fixation

In clinical practise, the shoulder positioning during ASCR is usually performed on the operating table using a lateral decubitus or beach chair positioning. This relates to the humerus being at 20°–45° of abduction, neutral abduction and flexion or 10° abduction with 70° forward flexion [2, 11]. The shoulder position of fixation defines the configuration of the shoulder girdle and the distance between origin and insertion sites of the graft. Graft fixations at low degrees of abduction produce higher reference lengths than fixations at high degrees of abduction. Smaller reference lengths produce deformations of the graft for a higher range of movement, and thus, more contribution of the hammock effect in centring the humeral head. For the same shoulder position of fixation, fixing the graft under tension relates to smaller reference lengths. Most of the biomechanical studies have tested graft fixation just considering GH abduction, rather than combined multiplanar GH fixation angle. In fact, most of the biomechanical studies used shoulder positions of fixation with 20° or 30° of GH abduction.

For FL grafts and a full‐thickness tear of the SSP, Mihata et al. [38] reported that fixations at both low (10°) and mid (30°) GH abduction angles effectively restored superior shoulder stability and restored subacromial contact pressure at 0°, 30° and 60° of abduction. Computational analysis, by Antunes et al. [7], suggested that fixation angles exceeding 15° of GH abduction predispose the graft to excessive strain when the arm returns to the resting position at the side of the trunk, which may lead to potential failure. For a series of shoulder motions evaluated, the most favourable position for shoulder stability was between 5° and 15° of abduction and 10° internal rotation. These findings indicate that moderate tensioning of FL grafts optimizes stability without overloading the tissue. Another computational study by Antunes et al. [6] indicated that when the tear extends anteriorly and/or posteriorly, there is a significant loss of stability independently of the shoulder position of fixation.

For HDA and a full‐thickness tear of the SSP, Adams et al. [1] reported that fixations at 15° of GH abduction recovered deltoid force levels of the intact condition for abduction in the scapular plane, while fixations at 60° of GH abduction significantly reduced deltoid force due to shorter grafts. These results were corroborated by Dyrna et al. [21], for which fixation of the graft at 30° of abduction, with a tensioned graft, correlates with increased maximal GH abduction (81% of the intact condition), while a non‐tensioned graft does not produce significant improvements (68% of the intact condition), compared to the tear state. For posterosuperior tears, Altintas et al. and Tibone et al. [3, 51] showed that HDA fixations at higher levels of abduction (40° or 45°) provided higher stability of the shoulder compared to fixations at 20° and 30°, respectively. Computational simulations by Hast et al. [27] supported these experimental findings, highlighting a 25° abduction and 20° internal rotation as an ideal configuration for optimal graft strain.

For LHBT grafts, Paccot et al. [45] found that native kinematics and functional abduction forces were best restored with fixations at 30° of abduction with either middle or posterior fixation of the LHBT in the greater tuberosity. Regarding the location of fixation, as a partial posterosuperior irreparable RCT was simulated, more posterior graft fixations restored better the anterior‐posterior stability.

### Graft thickness

The majority of the collected studies only evaluated the role of the graft as a spacer against superior translations and did not consider the contribution of the graft during shoulder motion for the stability of the GH joint.

For FL grafts, Mihata et al. [38] showed that an 8 mm thick graft restored superior GH stability more effectively than a 4 mm graft, suggesting that thicker grafts provide better mechanical resistance to superior translations. At 30° of abduction, the superior GH translation was 73% and 146% of the intact condition for the 8 and 4 mm graft, respectively. Nevertheless, Hu et al. [28] found that, for a partial posterosuperior tear, increasing thickness (from two‐ to four‐folds) improved humeral head recentring, but did not enhance GH range of motion (RoM) and even increased subacromial contact pressures.

The studies by Cline et al., Scheiderer et al. and Smith et al. [15, 47, 50] showed that 6‐mm or double‐layer HDA better restored stability and kinematics than 3‐mm or single‐layer grafts. To note that the thickness investigated in these studies remained below the recommended FL thickness.

### Fixation technique

The fixation technique has a wide variability, but a single row is commonly used on the glenoid rim, and a double row fixation is used on the humerus side [2]. The number and type of anchors fixed have an impact on the load distribution and may be responsible for anchor pull‐out and early failure of the graft. Most of the biomechanical studies focused on the glenoid side fixation as it has a single row and may present higher risk of anchor pull‐out. Schon et al. [48] reported that the fixation of three anchors in the glenoid side provided optimal fixation strength without compromising the suprascapular nerve pathway. Pogorzelski et al. [46] showed that fixing three threaded 3.5‐mm suture anchors presented a higher pull‐out strength than two 3.0‐mm knotted push‐in anchors combined with two 2.9‐mm knotless push‐in anchors. Dasari et al. [17] showed that a 4.5‐mm Bio‐Corkscrew anchor showed the strongest maximum load and minimal elongation, compared to the conventional 3.0‐mm knotless suture anchor (SutureTak) and 3.9‐mm knotless PEEK (polyetheretherketone) Corkscrew anchor. These results suggest that the anchor design and bone engagement play a more significant role in pull‐out strength than the number of anchors fixed. Also, Ting et al. [52] observed that minitape is not superior to suture in terms of pull‐out strength, in the case of two‐anchor repair.

## DISCUSSION

The main finding of this systematic review was that the graft type and dimensions influence the level of recovery of joint stability and kinematics, while the performance of concomitant procedures is required to reestablish the anterior‐posterior force couple, confirming our hypothesis. The majority of the biomechanical studies focused on the role of the graft as a spacer to counteract superior humeral head translation and to reduce subacromial contact pressures, while providing limited insights into its influence on the dynamic balance of the shoulder joint. The potential contribution of the graft to humeral head centring and to redistribution of muscle forces, among the remaining dynamic stabilizers, has been largely overlooked. This limitation is particularly relevant in conditions where the tendons of ISP and/or SSC are not repaired, as these scenarios emphasize that the graft alone may restore superior stability but is not able to restore anterior‐posterior stability loss, highlighting the importance of tendon repair to re‐establish force coupling.

Within the included studies, cadaver‐based experimental approaches predominated. The majority of the studies implemented static muscle loading, about 70% of the studies that considered muscle loading, often adopting the same combination of the muscle loads proposed by Mihata et al. [41]. The majority of the outcomes were evaluated at discrete GH joint configurations, most commonly at 0°, 30° and 60° of GH abduction. Although these simplifications facilitate experimental control, they limit the ability to reproduce functional shoulder biomechanics. In particular, they do not capture activities of the daily living (ADLs) that involve multiplanar motions and complex load‐sharing problems between muscles and graft. The only three studies that evaluated joint stability and/or joint kinematics for ADLs were the three computational studies, giving strength to the use of these methodological approaches [6, 7, 27].

Another important gap of the current literature is that most biomechanical studies investigated the biomechanical impact of a single surgical or graft‐related factor in isolation, with the exception of only four studies that evaluated the combined impact of graft type and thickness or fixation technique, graft thickness and shoulder position of fixation and concomitant tenotomy of the LHBT and the shoulder position of fixation [7, 15, 38, 52]. This approach, together with substantial heterogeneity in experimental protocols—including differences in tear definition, graft material and dimensions, fixation technique (when reported), muscle loading strategies and concomitant procedures (frequently not described)—limits the generalization of biomechanical findings and the direct comparison of results across studies.

Concomitant procedures comprised the largest group of factors evaluated in the literature regarding biomechanical studies of ASCR, reflecting the wide array of surgical strategies proposed to restore stability and force coupling, depending on patient‐specific pathology and surgeon preference. The selection and combination of these procedures reflect an effort to optimize joint biomechanics, reduce pain and enhance the functional integration of the graft [2, 14]. Concomitant procedures addressing the acromion highlighted a trade‐off between restoring superior translation and increasing acromion impingement, which could lead to graft tear and failure. While LHBT tenotomy or tenodesis is frequently performed concomitantly to ASCR, in most of the cadaveric studies, the performance of a concomitant tenotomy or tenodesis was not described.

With respect to graft‐related factors, FL autograft was originally proposed as the graft material for ASCR and has demonstrated favourable biomechanical performance in terms of restoring shoulder stability and kinematics [37, 41]. Overall, the biomechanical evidence indicates that while the FL remains the biomechanical gold standard, the HDA with suture‐tape reinforcement and specific configurations of the LHBT graft may provide comparable performance under specific conditions. However, HDAs have been reported to exhibit greater elongation under cyclic loading, when compared to FL autografts, suggesting that future biomechanical studies should explicitly account for time‐dependent deformation and elongation of the graft rather than relying solely on quasi‐static conditions [36]. The impact of graft thickness was primarily evaluated using metrics that define the role of the graft as a spacer, such as superior humeral translation and subacromial contact pressure; however, the influence of the thickness of the graft on the risk of graft tearing, from pressures with the acromion, has not been sufficiently explored. Such contact may contribute to postoperative shoulder pain and, ultimately, compromise the integrity of the graft and lead to graft failure.

The shoulder position during graft fixation is defined by the orientation of the humerus, described in terms of abduction, forward flexion and axial rotation. As the graft is typically secured without slack, this position establishes the reference length of the graft. The selected shoulder position, therefore, plays a critical role in defining the graft reference length and mechanical behaviour. Most biomechanical studies focused solely on the abduction angle, with fixation positions between 15° and 30° of GH abduction generally providing a compromise between graft tensioning and shoulder stability. Lower abduction angles risk insufficient tension, while higher abduction angles may lead to graft failure, with the optimal angle varying with the material's stiffness and elasticity. Three computational studies, with FL and HDA grafts, provided evidence that the flexion and axial rotation of the humerus also influence the reference length of the graft and should be incorporated in future biomechanical evaluations of ASCR [6, 7, 27].

In addition, studies examining fixation techniques evaluated joint‐level outcomes, with limited consideration of the interactions between anchors, sutures and grafts. The available studies relied mainly on load‐to‐failure of in vitro models, with or without the graft. Further research is required to study the dynamic interactions between anchors, suture and graft and prevent the early failure of the graft fixation, which may provide clinically relevant insights into failure mechanisms of ASCR, including the risk of anchor pull‐out and graft failure.

Taken together, these limitations highlight a substantial gap in the current biomechanical understanding of ASCR. Computational biomechanical frameworks emerge as a valuable complementary approach to in vitro studies, enabling controlled and systematic comparisons of surgical and graft‐related factors and their combined effects under reproducible conditions. Moreover, such methodological approach allows the estimation of outcome variables that are difficult or unfeasible to measure experimentally using in vivo or in vitro approaches, such as joint reaction forces, muscle activation patterns and graft strain distribution.

Despite the biomechanical evidence and the identification of knowledge gaps, the systematic review is not free of limitations. First, the substantial heterogeneity across included studies, regarding experimental setups, modelling assumptions, outcome measures and reported variables, prevented quantitative synthesis. Consequently, no meta‐analysis, subgroup analysis or sensitivity analysis was performed, and the findings are based on qualitative interpretation of the available evidence. Second, the included studies comprised experimental and computational biomechanical studies, for which no validated risk‐of‐bias assessment tool currently exists across all study types. A methodological quality assessment was performed for the 32 cadaveric studies included in this review using the QUACS scoring system. However, for the remaining in vitro and computational studies, risk‐of‐bias tools developed for clinical randomized or observational studies are not applicable. Consequently, methodological characteristics and potential sources of bias, such as specimen preparation, experimental protocols, loading conditions and modelling assumptions, were considered qualitatively when interpreting the results.

## CONCLUSION

The current biomechanical evidence on ASCR remains constrained by methodological simplifications and the isolated, rather than combined, assessment of ASCR‐related factors. Available data suggest that graft type and dimensions, and the performance of concomitant procedures restoring capsule continuity, influence the recovery of joint stability.

## AUTHOR CONTRIBUTIONS

Madalena Antunes conceived the idea for the review and defined the research objectives. Madalena Antunes performed the literature search, study selection and data extraction, with methodological input and verification provided by Ana Catarina Ângelo and Clara Isabel de Campos Azevedo. Data analysis and interpretation were conducted by Madalena Antunes in consultation with Ana Catarina Ângelo and Clara Isabel de Campos Azevedo. Madalena Antunes drafted the manuscript, and all authors critically revised the work for important intellectual content and approved the final version.

## FUNDING INFORMATION

FCT.IP; Associated Laboratory for Energy, Transport and Aeronautics, Grant/Award Number: UID.50022.2025; Fundação para a Ciência e a Tecnologia (FCT), Grant/Award Number: 2021.06844.BD.

## CONFLICT OF INTEREST STATEMENT

The authors declare no conflicts of interest.

## ETHICS STATEMENT

The authors have nothing to report.

## Supporting information

Appendix_1.

Appendix_2.

## Data Availability

Data sharing is not applicable to this article as no datasets were generated or analysed during the current study.
