# Supplementary material for: Biomechanical influence of surgical and graft‐related factors in superior capsule reconstruction: A systematic review
Source: J Exp Orthop. 2026 Jun 15;13(2):e70804. doi: 10.1002/jeo2.70804 (PMC13266580; doi:10.1002/jeo2.70804)
Supplement: Supplementary file 1 — Appendix_1. [file JEO2-13-e70804-s002.docx]

APPENDIX 1

Table A1 Summary of the complete search queries for each of the electronic databases used for the systematic review.

| **Eletronic Database** | **Search Query** |
| --- | --- |
| PubMed | (( AB=(superior AND (capsule OR capsular) AND reconstruction)) AND AB=(cadaver* OR biomechanic* OR robot* OR comput* OR in vivo OR in silico OR in vitro)) AND PY=(2012-2026) Refined by: “Languages: English” |
| Web of Science | ((superior[Title/Abstract]) AND (capsul*[Title/Abstract]) AND (reconstruction[Title/Abstract])) AND ((cadav*[Title/Abstract]) OR (biomechanic*[Title/Abstract]) OR (robot*[Title/Abstract]) OR (comput*[Title/Abstract]) OR (in vivo[Title/Abstract]) OR (in silico[Title/Abstract]) OR (in vitro[Title/Abstract]))  Filtered by: “Results by year: 2012-2026” and “Article Language: English” |
| Scopus | (TITLE-ABS-KEY ( "Superior Capsule Reconstruction" OR "Superior Capsular Reconstruction" ) ) AND ( TITLE-ABS-KEY ( cadaver* OR biomechanic* OR robot* OR comput* OR "in vitro" OR "in vivo" OR "in silico" ) ) Filtered by: “Results by year: 2012-2026” and “Language: English” |
| Cochrane Library | #1 ("Superior Capsule Reconstruction" OR "Superior Capsular Reconstruction" OR SCR):ti,ab,kw #2 (cadaver* OR biomechanic* OR robot* OR comput* OR "in vitro" OR "in vivo" OR "in silico"):ti,ab,kw #3 #1 AND #2  Filtered by: “Results by year: 2012-2026” and “Language: English ” |
| Ovid | (AllFields:((Abstract:(superior) AND (Abstract:(capsule) OR Abstract:(capsular)) AND Abstract:(reconstruction)) AND (cadaver* OR biomechanic* OR robot* OR computat* OR (in vivo) OR (in vitro) OR (in silico))))  Filtered by: “Results by year: 2012-2026” and “Language: English” |
