# Supplementary material for: Biomechanical influence of surgical and graft‐related factors in superior capsule reconstruction: A systematic review
Source: J Exp Orthop. 2026 Jun 15;13(2):e70804. doi: 10.1002/jeo2.70804 (PMC13266580; doi:10.1002/jeo2.70804)
Supplement: Supplementary file 2 — Appendix_2. [file JEO2-13-e70804-s001.docx]

APPENDIX 2

Table A2 Summary of the excluded studies after the full-text assessment for eligibility, grouped according to the respective exclusion criteria.

| **Exclusion criteria** | **References** |
| --- | --- |
| Editorials and Erratum | [18, 30, 36, 39, 45, 47, 52, 63, 66, 71, 78, 80, 91, 105–107, 124, 127, 129, 135, 139, 143, 169, 173–176, 181, 187, 189] |
| Surgical technique, indications to use ASCR, description of the surgical factors | [2, 3, 11, 23, 27, 28, 31, 40, 44, 48, 50, 51, 53–57, 64, 65, 69, 70, 72, 79, 94, 95, 98, 102, 103, 120, 121, 125, 126, 130–132, 138, 140, 144, 145, 148, 149, 153, 155, 157, 160, 162, 166, 167, 171, 182, 184, 185] |
| Clinical outcomes | [5, 13, 14, 17, 26, 46, 58, 59, 67, 74–77, 85, 86, 90, 92, 97, 100, 101, 104, 108, 116, 128, 134, 136, 137, 151, 156, 159, 161, 170, 188] |
| Animal Studies | [93, 168, 183] |
| Comparison with other techniques to treat IRCTs | [12, 15, 16, 41, 141, 152, 158] |
| No comparison of biomechanical outcomes of ASCR | [22, 32, 35, 49, 84, 96, 110, 115, 118, 142, 172] |
| Reviews | [99, 165, 186] |
